# Randomized Comparative Study Assessing the Efficacy of Intralesional Measles, Mumps, and Rubella (MMR) Vaccine and Vitamin D3 in Treating Warts

**DOI:** 10.7759/cureus.78337

**Published:** 2025-02-01

**Authors:** Sushantika Sushantika, Nripendra Singh

**Affiliations:** 1 Dermatology, All India Institute of Medical Sciences, Rishikesh, Rishikesh, IND; 2 Community Medicine, Maharaja Suhel Dev Autonomous State Medical College and Mahrishi Balark Hospitals, Bahraich, Bahraich, IND

**Keywords:** cutaneous warts, intralesional immunotherapy, measles mumps rubella (mmr), rct, vitamin d3

## Abstract

Background: Warts are benign skin growths caused by variants of human papillomavirus that infect the superficial layers of the skin and penetrate epithelial cells, causing viral multiplication. Warts can be transmitted via skin-to-skin contact and cause significant discomfort and embarrassment. There are multiple treatment options for warts, including topical therapies, cryotherapy, laser vaporization, surgical excision, and oral agents (e.g., zinc, retinoid, levamisole). In recent years, immunotherapy with various antigens has successfully treated multiple lesions by combining a targeted approach with upregulation of the host’s immune system. This study explores and compares the efficacy of two immunotherapy options to treat warts: vitamin D3 and the measles, mumps, and rubella (MMR) vaccine.

Methodology: A randomized prospective comparative study was conducted to determine the efficacy of intralesional vitamin D3 and MMR vaccine in patients suffering from warts. The study population was categorized into two groups. The patients were divided into two equal groups, Group A (MMR; n=90) and Group B (vitamin D3; n=90), using a computer-generated random number table after applying the allocation concealment method to segregate participants and then allocated into either group using the sequentially numbered, opaque, sealed envelope (SNOSE) technique. One group received intralesional vitamin D3, and the other received intralesional MMR vaccine. Researchers followed up with patients for three months after completion of their therapy to notice any relapse. The outcomes considered in our study were recovery (complete, partial, and no recovery) and side effects such as pain, burning sensation, and desquamation. The statistical analysis was done using IBM SPSS Statistics for Windows, Version 22 (Released 2013; IBM Corp., Armonk, New York, United States).

Results: At the end of the study, out of 150 subjects who reached the end, complete clearance was seen in 42/70 (60%) patients in Group A compared to only 26/80 (32.5%) patients showing clearance in Group B after completing six sessions of therapy in either group. Applying the chi-square test, the p-value comes to be 0.001323, so the result is significant at p<0.05. Hence, the MMR vaccine is better than an intralesional injection of vitamin D3 in treating warts.

Conclusion: Intralesional MMR vaccine showed a better response than injection of vitamin D3 in treating warts as an immunotherapeutic agent.

## Introduction

Human papillomavirus (HPV) consists of a family of small, double-stranded circular DNA viruses that infect the epithelium. More than 200 distinct types have been identified and differentiated on the basis of their genomic sequence. Most HPV virus types infect cutaneous or mucosal epithelium and cause common skin warts, leading to cosmetic disfigurement and pain [1,2]. The average prevalence of warts ranges from 7% to 12% of the population, especially in school-going children, immunocompromised individuals, and people who handle meat. Spontaneous remission of warts is noted in two-thirds of children within two years. In adults, remission tends to be slower and may take up to four to five years or longer [3,4]. Clinical presentation of warts includes flat warts (verruca plana), common warts (verruca vulgaris), plantar warts (verruca plantaris on the soles of feet), and condyloma acuminatum (anogenital warts) [5]. Multiple options are available to treat warts, including topical, systemic, and destructive procedures and immunotherapeutic options, depending on the warts’ location and refractory nature [6,7].

Immunotherapy for warts has been performed with diphenylcyclopropenone (DCP), squaric acid dibutyl ester (SADBE), imiquimod, tuberculin jelly, and autologous vaccines. The first contact allergen used to treat resistant warts was dinitrochlorobenzene (DNCB) in 1973. Recalcitrant, recurrent, or extensive warts, as well as warts affecting periungual and palmoplantar sites, are considered for immunotherapy. Intralesional immunotherapy utilizes the immune system’s ability to mount a delayed hypersensitivity response to various antigens and wart tissue. This therapy has been associated with the production of Th1 cytokines, which activate cytotoxic and natural killer cells to eradicate HPV infections. Unlike traditional therapies, this treatment clears distant warts in addition to local warts [8-10].

Vitamin D is a fat-soluble steroid prohormone that has endocrine, paracrine, and intracrine functions. It has a wide range of biological actions (e.g., inhibition of cellular proliferation, induction of terminal differentiation, and inhibition of angiogenesis) and is available in two forms: vitamin D2, which is found in plants, and vitamin D3, which is found in food and produced by the cutaneous conversion of 7-dehydrocholesterol under the action of ultraviolet B [11,12]. No definite mechanism of action has been elucidated until now. However, with intralesional injection of vitamin D3, it is plausible that a toll-like receptor can activate human macrophages and upregulate the expression of vitamin D receptor (VDR) and vitamin D-1-hydroxylase genes, leading to the induction of the antimicrobial peptide and elimination of warts [13].

Alternatively, the measles, mumps, and rubella (MMR) vaccine, a freeze-dried preparation of live attenuated strains of measles, mumps, and rubella viruses, has been employed as an intralesional immunotherapy agent for warts because it mounts a Th1 immune response, increasing the production of TNF α, IL-2, IL-4, IL-5, and IFN-γ, and propagates a delayed hypersensitivity reaction against both MMR viral antigens and HPV [14,15].

Although a well-proven mechanism of action of immunotherapy agents is still enigmatic, their efficacy has been promising enough to explore further wart management over the course of years. This study was conducted with the objective of comparing and assessing the efficacy of intralesional vitamin D3 and MMR vaccines in treating warts.

## Materials and methods

Patient selection

In this prospective randomized comparative study, using the Cochrane formula, 180 was the total sample size. However, only 150 patients completed the study. The study was conducted in the Department of Dermatology, Venereology, and Leprosy at Maharaja Suhel Dev Autonomous State Medical College in Bahraich, Uttar Pradesh.

**Figure 1:**
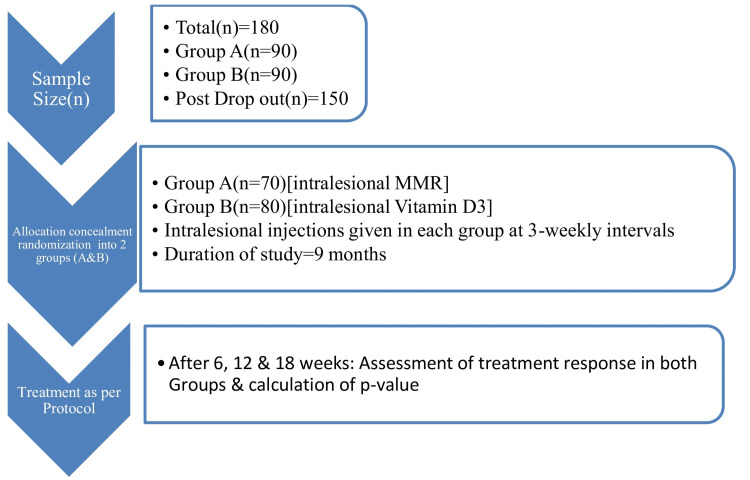
Flowchart of the study plan MMR: measles, mumps, and rubella

Male and female patients older than 12 years with 15 or fewer lesions of common warts, palmoplantar or peri-ungual warts, or genital warts were included in the study. The participants gave written consent for the immunotherapy. Patients who had received any topical or systemic therapy in the last three weeks, pregnant or lactating women, and immunocompromised patients were excluded. Additionally, patients on anticoagulant therapy, with a history of recent live vaccinations, or with any history of hypersensitivity to the agents proposed to be used for the study were excluded. The institutional ethical committee approved the study (Reference No: MSDAMC&MBH/BRH/2022-23/01A), and before the administration of injections, written informed consent was taken from all patients with their signatures. The patients were divided into two equal groups, Group A (MMR; n=90) and Group B (vitamin D3; n=90), using a computer-generated random number table after applying the allocation concealment method to segregate participants and then allocated into either group using the sequentially numbered, opaque, sealed envelope (SNOSE) technique (Figure 1).

Treatment protocol

The procedure was done in the minor OT in the Dermatology Department at the Maharaja Suhel Dev Autonomous State Medical College, and patient data was incorporated in a Microsoft Excel (Microsoft Corporation, Redmond, Washington, United States) for all subjects so that the statistical analysis could be performed easily at the end of the study. As the primary investigator of the study had to shift from the college to All India Institute of Medical Sciences (AIIMS), Rishikesh, after completing the study, data was stored safely in Excel for statistical analysis done by the investigator based in Bahraich itself.

Group A patients were treated with intralesional MMR vaccine (freeze-dried rubella vaccine (1000.0 Ccid) + mumps vaccine (5000.0 Ccid) + measles vaccine (1000.0 Ccid), brand: Tresivac 0.5 ml (Serum Institute of India Pvt. Ltd., Pune, India), where 0.3 ml of the vaccine was injected in the base of the largest wart, and the rest were treated with 0.1 ml vaccine injections with an insulin syringe. The vaccine is diluted with 0.5 ml of sterile water provided with the vaccine before injection, which was repeated every three weeks for up to six sessions or until all the warts had cleared.

Group B patients were injected with a 0.2- to 0.5-mL vitamin D3 solution (600,000 IU, 15 mg/mL, brand: Arachitol 6 L (Abbott, Mumbai, India)) at the base of the wart with a 3 ml syringe every three weeks for up to six sessions or until all the warts had cleared. Up to five warts were injected at a time. To avoid painful injections, lignocaine was injected at the wart site prior to the injection of the vitamin D3 solution in patients who could not tolerate plain injections.

Assessment of response

The number and distribution of warts and local side effects were noted at six, 12, and 18 weeks. A continuous clinical photographic record was maintained. Responses were evaluated as follows: a complete response was the clearance of all warts completely; a partial response was a reduction in the number or size of warts; and no response was a negligible reduction in the number and size of warts. Adverse effects (constitutional or local symptoms, if present) were noted. Researchers followed up with patients who showed complete responses for three months to note any recurrence or residual skin changes. The collected data was analyzed according to the statistical plan.

Statistical analysis

Therapeutic responses at different time points were compared between two treatment modalities using the chi-square test. The types of warts were reported in two treatment modalities across the therapeutic response. The p-value <0.05 was considered statistically significant. The statistical analysis was done using IBM SPSS Statistics for Windows, Version 22 (Released 2013; IBM Corp., Armonk, New York, United States).

## Results

A total of 150 subjects completed the study after dropouts were excluded. Of those who completed the study, 70 patients received an intralesional MMR vaccine, and 80 received an intralesional injection of vitamin D3. About 80% of participants were less than 35 years of age. About 57.33% were male, which may be due to greater acquisition of disease because of greater indulgence in outdoor activities and contact with different people (Table 1). About 56.67% of the subjects in our study had palmoplantar warts, followed by verruca vulgaris (18.67%), filiform warts (16%), and genital warts (8.67%) (Table 2).

**Table 1:** Demographic details of the study population MMR: measles, mumps, and rubella

Patient Characteristics	Group A/MMR vaccine(n=70)	Group B/Injection Vitamin D3 (n=80)
Age of subjects in years		
12–20	20	28
20–35	40	32
35–60	10	20
Sex of subjects		
Male	40	46
Female	30	34
Residence		
Rural	53	58
Urban	17	22

**Table 2:** Types of warts being treated in this study MMR: measles, mumps, and rubella

Types of Warts	GroupA/MMR vaccine(n= 70)	GroupB/Injection Vitamin D3(n=80)
Verruca vulgaris	12 (17.1%)	16 (20%)
Filiform	9 (12.86%)	15 (18.75%)
Palmar	23 (32.86%)	20 (25%)
Plantar	20 (28.57%)	22 (27.5%)
Genital	6 (8.57%)	7 (8.75%)

**Figure 2:**
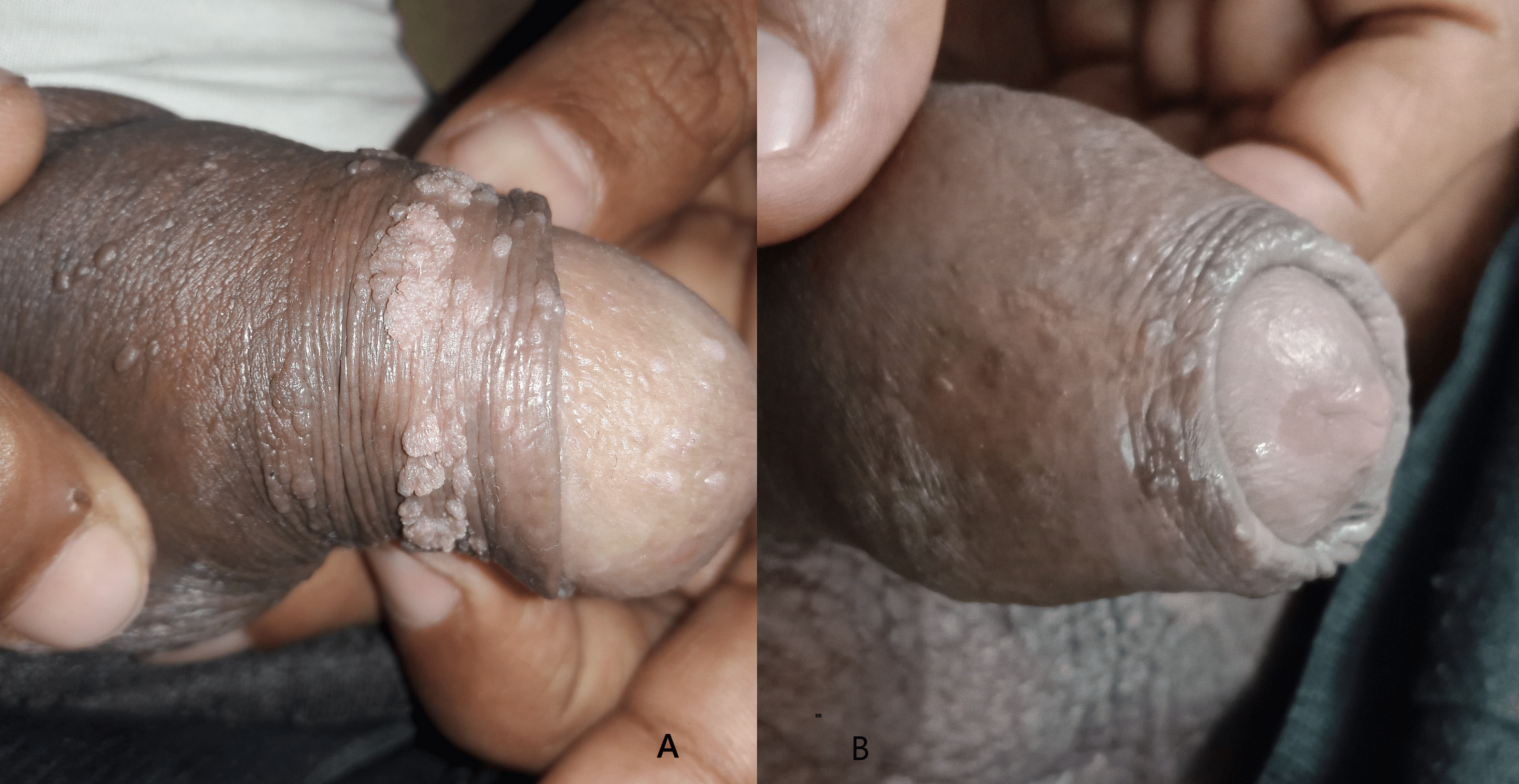
Complete clearance at the end of 18 weeks with intralesional MMR in genital warts MMR: measles, mumps, and rubella

**Figure 3:**
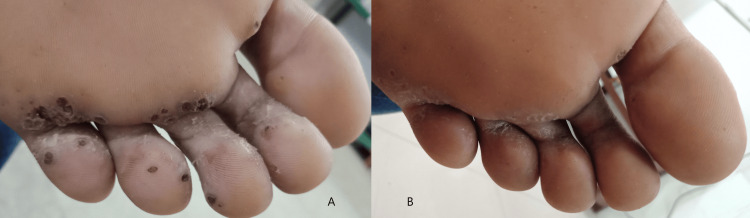
Complete clearance noted with intralesional MMR vaccine in plantar warts at the end of 12 weeks MMR: measles, mumps, and rubella

**Figure 4:**
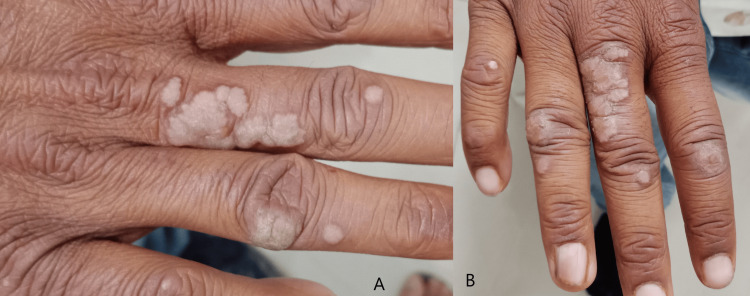
Partial response was seen with intralesional vitamin D3 injection on verruca vulgaris at the end of 18 weeks

**Figure 5:**
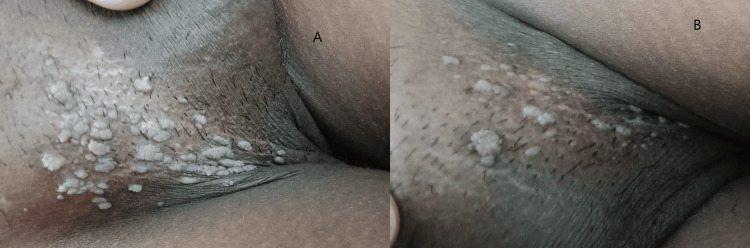
Partial response at the end of 18 weeks in genital warts with intralesional vitamin D3

At six weeks, 64.3% of the subjects in Group A had less than a 50% reduction in size and area of warts, and in Group B, 60% of subjects had a similar response. At 12 weeks, around 14% of subjects in Group A had a complete response to therapy, but only 5% of subjects had a complete resolution in Group B. At the end of 18 weeks of therapy, 60% of subjects in Group A had complete clearance of warts, but in Group B, 32.5% of subjects had a complete clearance of warts (Table 3). These results show that the MMR vaccine is significantly more effective than the injection of vitamin D3 in clearing warts (Table 4). Figures 2, 3, 4, 5 depict the response seen in both groups of a few patients.

**Table 3:** Treatment response at various weeks MMR: measles, mumps, and rubella

Duration	Group A(MMR) (n=70)	Group B (Vitamin D3) (n=80)
6 weeks	Partial response: 45 (64.3%), no response: 25 (35.7%), complete response: 0	Partial response: 48 (60%), no response: 32 (40%), complete response: 0
12 weeks	Partial response: 56 (80%), no response: 4 (5.7%), complete response: 10 (14.3%)	Partial response: 60 (75%), no response: 16 (20%), complete response: 4 (5%)
18 weeks	Partial response: 26 (37.14%), no response: 2 (2.86%), complete response: 42 (60%)	Partial response: 40 (50%), no response: 14 (17.5%), complete response: 26 (32.5%)

**Table 4:** Outcomes and results at the completion of the study MMR: measles, mumps, and rubella

Immunotherapy Used (Intralesional)	Cured	Uncured	Total	Cure Rate
MMR vaccine	42	28	70	60%
Vitamin D3	26	54	80	32.5%
*The corrected chi-square statistic comes to be 10.3099, and the p-value is 0.001323, which is significant at p<0.05.				

In both groups, pain was the most common side effect noted. However, with injected vitamin D3, almost 95% of subjects experienced pain, and with the MMR vaccine, around 60% of patients had this complaint. Other side effects were erythema and swelling for one to two hours after therapy, slightly more with vitamin D3 injection. None of the subjects developed any hypersensitivity reactions to either of the agents.

## Discussion

With the moderately high prevalence and resistant nature of warts, dermatologists must find new ways to treat this ordinary viral infection.

This study showed that the intralesional MMR vaccine has better efficacy and a more favorable side effect profile than intralesional vitamin D3 in treating warts. By the end of this study, 60% of the subjects in Group A (MMR) had complete clearance of warts, and 32.5% of the subjects in Group B (vitamin D3) had a complete clearance of warts. As for the side effect profile, around 95% of the subjects reported pain in the vitamin D3 group, and around 60% reported pain in the MMR group. A burning sensation was more pronounced in the MMR group, and persistent edema at the injection site was more apparent in the vitamin D3 group. The better responses seen with MMR may be due to the generation of HPV-directed immunity via the induction of a delayed-type hypersensitivity reaction to an otherwise unrelated immunogen in warts both at the site of injection and for distant lesions.

In a similar study conducted by Singh A et al., complete response was seen in 54% and 62% of study participants in the vitamin D3 and MMR groups, respectively, suggesting that MMR was slightly superior to vitamin D3 in terms of warts clearance and side effects profile [16].

In a similar study by Jartarkar Shishira et al., 52% (17) of people in the vitamin D3 group got rid of all of their warts, while 70% (23) of people in the MMR group did the same. An excellent complete response was noted in 82% (27) of participants with vitamin D3 and 91% (30) of cases with the MMR vaccine [17]. The clinical improvement noted with the intralesional MMR vaccine was not statistically different than with intralesional vitamin D3 (P > 0.05).

In a study conducted by Ahmed Jafir et al. on pediatric cases of warts, a complete response in injected warts in the MMR group was seen in 25 (83.3%) patients, a partial response in three (10%), and no or inadequate response in two (6.67%). In the vitamin D3 group, complete response was seen in 23 (76.66%) patients, partial response in four (13.33%), and no or inadequate response in three (10%) patients. Complete response was seen in distant warts in 73.33% of patients with MMR and 66.66% of patients with vitamin D3 [18].

In all these similar studies, we observe that the MMR vaccine and vitamin D3 are almost on par with each other in the treatment of recalcitrant warts. However, as per our study, the MMR vaccine does have marginally better efficacy than vitamin D3 in warts clearance. Being a randomized controlled study was a benefit of this study, but multi-institutional studies with larger sample sizes would be needed to decide which immunotherapeutic agent is better for managing warts.

## Conclusions

New immunotherapeutic agents are constantly being explored for the treatment of recalcitrant and recurrent warts. Traditional modalities of destruction are not always feasible for patients, and systemic therapy typically takes a long time to achieve the desired response. This study is an extension of the implications of using two of the available agents to treat warts. If cost is not an issue, then MMR seems to be more effective at eliminating warts. Otherwise, vitamin D3 performs at an acceptable standard.

## References

[1] Lyon (FR) (2007). IARC Monographs on the Evaluation of Carcinogenic Risks to Humans. https://www.ncbi.nlm.nih.gov/books/NBK321760/.

[2] Zheng Zheng, ZM ZM (2013). Human Papillomavirus (HPV). Encyclopedia of AIDS.

[3] Al Aboud AM, Nigam PK (2025). Wart. StatPearls [Internet].

[4] Essa N, Saleh MA, Mostafa RM, Taha EA, Ismail TA (2019). Prevalence and factors associated with warts in primary school children in Tema District, Sohag Governorate, Egypt. J Egypt Public Health Assoc.

[5] Zhu P, Qi RQ, Yang Y (2022). Clinical guideline for the diagnosis and treatment of cutaneous warts (2022). J Evid Based Med.

[6] Bacelieri R, Johnson SM (2005). Cutaneous warts: an evidence-based approach to therapy. Am Fam Physician.

[7] Stulberg DL, Hutchinson AG (2003). Molluscum contagiosum and warts. Am Fam Physician.

[8] Chandrashekar L (2011). Intralesional immunotherapy for the management of warts. Indian J Dermatol Venereol Leprol.

[9] Choi Y, Kim DH, Jin SY, Lee AY, Lee SH (2013). Topical immunotherapy with diphenylcyclopropenone is effective and preferred in the treatment of periungual warts. Ann Dermatol.

[10] Chandran B (2022). Topical and intralesional immunotherapy in cutaneous infections. J Skin Sex Transm Dis.

[11] Bikle DD (2000). Vitamin D: Production, Metabolism and Mechanisms of Action. Endotext [Internet].

[12] Liu PT, Stenger S, Li H (2006). Toll-like receptor triggering of a vitamin D-mediated human antimicrobial response. Science.

[13] Al-Sabak H, Al-Hattab M, Al-Rammahi M, Al-Dhalimi M (2023). The efficacy of intralesional vitamin D3 injection in the treatment of cutaneous warts: a clinical therapeutic trial study. Skin Res Technol.

[14] Rezai MS, Ghasempouri H, Asqary Marzidareh O Yazdani Cherati J, Rahmatpour Rokni G (2019). Intralesional injection of the measles-mumps-rubella vaccine into resistant palmoplantar warts: a randomized controlled trial. Iran J Med Sci.

[15] Saini P, Mittal A, Gupta LK, Khare AK, Mehta S (2016). Intralesional mumps, measles and rubella vaccine in the treatment of cutaneous warts. Indian J Dermatol Venereol Leprol.

[16] Singh AK, Devi KN, Bist JS (2022). A comparative evaluation of therapeutic response in warts to intralesional vitamin D3 versus intralesional measles, mumps, and rubella vaccine. Dermatol Ther.

[17] Shishira J, Manjunath K, Mamatha P, Swayam M, Spoorthy B (2021). A comparative study of therapeutic efficacy of intralesional measles, mumps, and rubella vaccine and intralesional vitamin D3 in the treatment of recurrent warts. Jour of Dermatol and Dermatol Surg.

[18] Ahmed J, Kushwaha RK, Sharma A, Agarwal M, Sharma N, Jain SK (2023). Comparison of the efficacy of intralesional measles, mumps, and rubella vaccine with intralesional vitamin D3 for the treatment of extragenital warts in pediatric age group (5-18 years). Ind Journ of Paed Dermatol.

